# Immature granulocytes and neutrophil-to-lymphocyte ratio as markers of treatment response in subacute thyroiditis

**DOI:** 10.20945/2359-4292-2023-0012

**Published:** 2024-06-19

**Authors:** Emek Topuz, Dilek Tüzün, Murat Şahin

**Affiliations:** 1 Necip Fazıl City Hospital Kahramanmaraş Turkey Endocrinology and Metabolism Specialists, Kahramanmaraş, Necip Fazıl City Hospital Kahramanmaraş, Turkey; 2 Division of Endocrinology and Metabolism Kahramanmaraş Sütçü Iman University Faculty of Medicine Kahramanmaraş Turkey Division of Endocrinology and Metabolism, Kahramanmaraş Sütçü Iman University Faculty of Medicine, Kahramanmaraş, Turkey

**Keywords:** Subacute thyroiditis, neutrophil to lymphocyte ratio, immature granulocyte

## Abstract

**Objective:**

Subacute thyroiditis is also known as subacute granulomatous thyroiditis, giant cell thyroiditis, painful thyroiditis, and De Quervain’s thyroiditis. Immature granulocytes (IG) and neutrophil-to-lymphocyte ratio (NLR) are new inflammatory markers that are easily detected in routine complete blood count (CBC) tests. The aim of this study was to investigate the role of IG and NLR as markers of treatment response in patients with subacute thyroiditis.

**Subjects and methods:**

The study included 41 patients with subacute thyroiditis treated and monitored in our outpatient clinic between April 2020 and April 2022. From a retrospective review of medical records, we recorded results of IG, NLR, thyroid-stimulating hormone (TSH), free thyroxine (fT4), free triiodothyronine (fT3), erythrocyte sedimentation rate (ESR), and C-reactive protein (CRP) from blood tests obtained routinely before and after treatment.

**Results:**

Overall, 31 (75.6%) patients were women and 10 (21.4%) were men. The median age was 41 years (range 22-68 years). The laboratory tests showed the following median (range) results: IG, 0.03 (0.01-0.08); NLR, 3.6 (1.2-5.2); TSH, 0.02 mIU/L (0.01-3.35 mIU/L); fT4, 2.3 ng/dL (1.0-7.0 ng/dL); fT3, 5.6 pmol/L (2.6-15.2 pmol/L); ESR, 49 mm/h (17.0-87 mm/h); and CRP, 73 mg/dL (3.0-188 mg/dL).

**Conclusion:**

Early diagnosis and treatment of subacute thyroiditis is fundamental. In the present study, the new inflammatory markers IG and NLR, measured routinely on CBC tests, decreased significantly after subacute thyroiditis treatment relative to pretreatment values. After treatment, the NLR change correlated with ESR and CRP changes, while the IG change correlated only with CRP change. These findings suggest that the markers IG and NLR may be used to evaluate treatment response in patients with subacute thyroiditis.

## INTRODUCTION

Subacute thyroiditis (also known as subacute granulomatous thyroiditis, giant cell thyroiditis, painful thyroiditis, or De Quervain’s thyroiditis) is a rare cause of thyrotoxicosis that affects women more often than men (3-5:1 ratio) (1). Patients with subacute thyroiditis present with neck pain and describe pain or tenderness on neck palpation. On thyroid function tests, these patients progress from thyrotoxicosis to euthyroidism and transient hypothyroidism before becoming euthyroid again. These phases are observed in only 40% of the patients; in 60% of them, the disease has a mild progression, without transient hypothyroidism or thyrotoxicosis, with patients remaining euthyroid. Permanent hypothyroidism develops in 5% of the patients (2).

Subacute thyroiditis is thought to result from an inflammatory process following a viral infection. Indeed, many patients have a history of upper respiratory tract infection about 2-8 weeks before symptom onset. Cases of subacute thyroiditis related to mumps, measles, adenovirus, Coxsackievirus, and SARS-CoV-2 infection have been reported in recent years (1,3-5).

In addition to thyroid pain and tenderness, almost all patients with subacute thyroiditis experience thyrotoxicosis in the early stages of the disease. Erythrocyte sedimentation rate (ESR) and C-reactive protein (CRP) values are high in the acute phase of the disease. Usually, ESR is greater than 50 mm/hour and, in some cases, may exceed 100 mm/hour (6).

Radioactive iodine uptake is low in the thyrotoxic phase of subacute thyroiditis (usually below 1%-3%). On ultrasound, the thyroid gland may be normal or hyperplastic, and diffuse or focal hypoechoic patchy areas may be seen. These areas are markedly painful when pressure from the ultrasound probe is applied. On color Doppler, a low flow is seen in the thyrotoxic phase (7).

Treatment may not be necessary in patients with mild symptoms of subacute thyroiditis, while symptomatic cases may be treated with nonsteroidal antiinflammatory drugs (NSAIDs) or corticosteroids. The latter is required in most cases due to the superior efficacy of prednisolone over NSAIDs in achieving symptom relief. Notably, the time to euthyroidism is similar with both treatments (8).

Increased levels of circulating immature granulocytes (IG), also known as left shift, reflect an increased IG to total granulocyte ratio or increased band neutrophil count. In sepsis, a left shift indicates the presence of a severe disease course. The delta neutrophil index (DNI) is the IG fraction determined by subtracting the fraction of mature polymorphonuclear leukocytes from the sum of myeloperoxidase-positive cells, reflecting the IG count (9). Both DNI and IG count increase in inflammatory and infectious events, indicating an active bone marrow (10).

The neutrophil-to-lymphocyte ratio (NLR) is an inflammatory marker easily calculated from complete blood count (CBC) tests. Many studies have associated this inflammatory marker with complications and prognosis of different diseases, including diabetic nephropathy (11), ischemic heart disease (12), and malignancies (13). The use of NLR in differentiating thyroid malignancy and nodular goiter has also been investigated (14).

The role of IG and NLR values in evaluating treatment response in patients with subacute thyroiditis has not been investigated in the literature. Thus, the aim of the present study was to evaluate the use of these markers in assessing treatment response in patients with subacute thyroiditis.

## SUBJECTS AND METHODS

The study protocol was approved by the institutional ethics committee (May 25, 2022, Session 2022/10, Decision number 2).

The present study included 41 patients with subacute thyroiditis treated and monitored in our hospital’s endocrinology outpatient clinic between April 2020 and April 2022. The diagnosis of subacute thyroiditis was based on history, physical examination, and clinical and laboratory/imaging findings.

Information on age, sex, comorbidities, use of medications, and results from CBC and thyroid ultrasound and scintigraphy were obtained from the patients’ medical files and hospital records. Additionally, IG, NLR, thyroid stimulating hormone (TSH), free thyroxine (fT4), free triiodothyronine (fT3), ESR, and CRP results obtained routinely before and after treatment were recorded. White blood cell, neutrophil, lymphocyte, and IG counts were measured using an automated hematology analyzer (XN3000; Sysmex Corp., Kobe, Japan), while NLR was calculated manually. Thyroid function tests (TSH, fT4, and fT3) were measured using commercially available kits and electrochemiluminescence immunoassay (Elecsys and Cobas 8000, Roche Diagnostics, Mannheim, Germany), while ESR was measured using an automated ESR analyzer (ESR-Vision, Shenzhen YHLO Biotech Co., Ltd., China) and CRP using nephelometry. None of the 41 patients included in the study had comorbidities (*e.g*., active or chronic infection, rheumatologic diseases, chronic kidney failure, malignancy, or chronic obstructive pulmonary disease) that could adversely affect the course and results of the study.

Thyroid function tests, ESR, CRP, IG, and NLR were measured at diagnosis (before treatment and during active disease). Depending on clinical findings and disease activity, NSAIDs or corticosteroids were prescribed. Patients given corticosteroids were prescribed prednisolone 40 mg/day, which was tapered weekly and discontinued after 6 weeks. Thyroid function tests, ESR, CRP, IG, and NLR, measured again 3 months after treatment, were recorded.

### Statistical analysis

A mean and standard deviation IG value of 0.02 before and after treatment was considered for sample size calculation. The calculated mean IG concentration after treatment was 0.02, and the standard deviation was 0.01. After initially considering an effect size of 1.15, which was relatively high, we ultimately established a value of 0.5. Assuming a power of 99%, an alpha error of 0.05, and an effect size of 0.5, the estimated sample size was 82.

The data are presented using descriptive statistics (mean, standard deviation, median, minimum–maximum, frequency, and ratio). Normality was tested with the Kolmogorov-Smirnov test. The nonparametric tests Mann-Whitney U test and Wilcoxon test were used for data with nonnormal distribution, and the parametric tests independent samples *t* test and paired samples *t* tests were used for data with normal distribution. The independent samples *t* test and Mann-Whitney U test were used to analyze independent quantitative data, and the paired samples *t* test and Wilcoxon test were used to analyze dependent quantitative data. Correlation analysis was done using Spearman’s correlation.

The statistical analysis was performed with SPSS, version 28.0 (IBM Corp., Armonk, NY, USA). P values < 0.05 were considered statistically significant.

## RESULTS

Of 41 patients included in the study, 31 (75.6%) were women and 10 (21.4%) were men. The median age was 41 years (range 22-68 years) (Table 1). All patients had severe pain on thyroid palpation. Overall, 10 patients (24.4%) had COVID-19 the month before the diagnosis of subacute thyroiditis, while 9 patients (22%) had viral infections other than COVID-19. Two patients (4.9%) had received a COVID-19 vaccine (both BioNTech) the month before the diagnosis of subacute thyroiditis.

**Table 1:** Characteristics of the study patients

Characteristics	Results
Age	41 (22-68), 41.5 ± 8.6
Sex	
Female	31 (75.6%)
Male	10 (24.4%)
RAIU (% uptake)	0.10 (0.01-2.80), 0.33 ± 0.75
TSH (mIU/L)	0.02 (0.01-3.35), 0.36 ± 0.81
fT4 (ng/dL)	2.3 (1.0-7.0), 2.5 ± 1.2
fT3 (pmol/L)	5.6 (2.6-15.2), 6.2 ± 2.7
ESR (mm/h)	49.0 (17.0-87.0), 50.7 ± 19.2
CRP (mg/dL)	73.0 (3.0-188.0), 75.6 ± 51.6
NLR	3.6 (1.2-5.2), 3.3 ± 1.1
IG	0.03 (0.01-0.08), 0.04 ± 0.02
Pain	41 (100.0%)
COVID-19 in the previous month	10 (24.4%)
No COVID-19 in the previous month	9 (22.0%)
COVID-19 vaccine in the previous month	2 (4.9%)
Site of inflammation (ultrasound)	
Right lobe	12 (29.3%)
Left lobe	2 (4.9%)
Both lobes	27 (65.9%)
Development of hypothyroidism	9 (22.0%)
Use of levothyroxine	6 (14.6%)
Corticosteroid treatment	25 (61.0%)
NSAID treatment	16 (39.0%)

The results are shown as numbers (percentages), median (minimum-maximum) values, or mean ± standard deviation values. Abbreviations: CRP, C-reactive protein; ESR, erythrocyte sedimentation rate; fT3, free triiodothyronine; fT4, free thyroxine; IG, immature granulocytes; NLR, neutrophil-to-lymphocyte ratio; NSAIDs, nonsteroidal antiinflammatory drugs; RAIU, radioiodine uptake; TSH, thyroid-stimulating hormone.

Thyroid ultrasound obtained at diagnosis revealed hypoechoic patchy areas distributed diffusely in the right lobe in 12 patients (29.3%), left lobe in 2 patients (4.9%), and bilaterally in 27 patients (64.9%). In all patients, color Doppler showed low flow. Thyroid scintigraphy, obtained at diagnosis in all patients, showed a median uptake level of 0.1% (0.01–2.80%). Laboratory test results showed the following median values: IG, 0.03 (0.01–0.08); NLR, 3.6 (1.2–5.2); TSH, 0.02 mIU/L (0.01–3.35 mIU/L); fT4, 2.3 ng/dL (1.0–7.0 ng/dL); fT3, 5.6 pmol/L (2.6–15.2 pmol/L); ESR, 49 mm/h (17.0–87 mm/h); and CRP, 73 mg/dL (3.0–188 mg/dL). Nine patients (22%) developed transient hypothyroidism after subacute thyroiditis, while 6 (14.6%) developed permanent hypothyroidism and required levothyroxine (Table 1).

**Figure 1:**
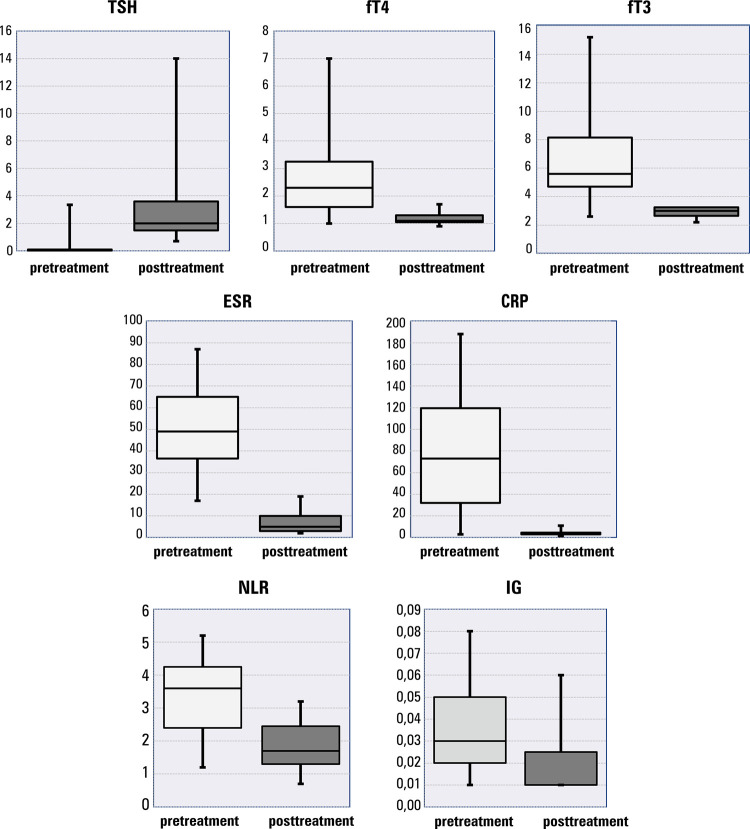
Changes in levels of thyroid-stimulating hormone, free thyroxine, free triiodothyronine, erythrocyte sedimentation rate, C-reactive protein, neutrophil-to-lymphocyte ratio, and immature granulocytes before and after treatment

Abbreviations: CRP, C-reactive protein; fT3, free triiodothyronine; fT4, free thyroxine; IG, immature granulocytes; NLR, neutrophil-to-lymphocyte ratio; TSH, thyroid-stimulating hormone.

After treatment, TSH values increased, and fT4 and fT3 values decreased in relation to pretreatment values (p < 0.05 for all). Posttreatment ESR, CRP, NLR, and IG values were significantly lower than those obtained before treatment (p < 0.05) **(**Figure 1 and Table 2).

**Table 2:** Comparison between pretreatment and posttreatment values of different tests

	Pretreatment				Posttreatment				p
	
	Min-max			Median	Min-max			Median	
TSH (mIU/L)	0.0	–	3.4	0.0	0.7	–	14	2.00	<0.001*
fT4 (ng/dL)	1.0	–	7.0	2.3	0.9	–	1.7	1.1	<0.001*
fT3 (pmol/L)	2.6	–	15.2	5.6	2.2	–	3.8	3.0	<0.001*
ESR (mm/h)	17	–	87	49.0	2.0	–	19	5.0	<0.001*
CRP (mg/dL)	3.0	–	188	73.0	1.4	–	11	3.2	<0.001*
NLR	1.2	–	5.2	3.6	0.7	–	3.2	1.7	<0.001*
IG	0.01	–	0.08	0.03	0.01	–	0.06	0.01	<0.001*

*Wilcoxon test. Abbreviations: CRP, C-reactive protein; fT3, free triiodothyronine; fT4, free thyroxine; IG, immature granulocytes; NLR, neutrophil-to-lymphocyte ratio; TSH, thyroid-stimulating hormone.

Values of ESR, CRP, and NLR decreased significantly after treatment in relation to pretreatment values, both in the NSAID and corticosteroid treatment groups (p < 0.05 for all). The pretreatment and posttreatment ESR, CRP, NLR, and IG values, as well as the changes in these values after treatment, were comparable between treatment groups (p > 0.05 for all) (Table 3).

**Table 3:** Comparison between pretreatment and posttreatment laboratory findings in patients treated with nonsteroidal antiinflammatory drugs versus corticosteroids

	NSAID treatment		Corticosteroid treatment		P values
	
	Mean ± SD	Median	Mean ± SD	Median	
TSH					
Pretreatment	0.46 ± 0.84	0.03	0.30 ± 0.81	0.02	0.879^‡^
Posttreatment	3.10 ± 3.13	2.06	2.89 ± 2.13	2.00	0.989^‡^
Change^*^	2.64 ± 3.45	1.87	2.59 ± 2.17	1.91	0.810^‡^
P values^†^	**0.003^¶^**		**<0.001^¶^**		
fT4					
Pretreatment	2.25 ± 0.98	2.25	2.72 ± 1.34	2.37	0.279^‡^
Posttreatment	1.19 ± 0.19	1.15	1.17 ± 0.17	1.10	0.702^‡^
Change^*^	-1.06 ± 0.98	-0.80	-1.55 ± 1.30	-1.27	0.095^‡^
P values^†^	**0.001^¶^**		**<0.001^¶^**		
fT3					
Pretreatment	5.4 ± 2.4	5.3	6.8 ± 2.7	6.0	0.068^‡^
Posttreatment	2.8 ± 0.3	2.8	3.0 ± 0.4	3.1	0.177^‡^
Change^*^	-2.5 ± 2.4	-2.5	-3.7 ± 2.7	-2.8	0.925^‡^
P values^†^	**0.001^¶^**		**<0.001^¶^**		
ESR					
Pretreatment	49.1 ± 16.5	50.0	51.6 ± 21.0	45.0	0.925^‡^
Posttreatment	7.4 ± 4.6	5.5	5.7 ± 3.2	4.0	0.165^‡^
Change^*^	-41.8 ± 16.0	-42.0	-45.9 ± 20.7	-40.0	0.584^‡^
P values^†^	**<0.001^¶^**		**<0.001^¶^**		
CRP					
Pretreatment	64.6 ± 49.9	64.0	82.6 ± 52.4	75.0	0.285^‡^
Posttreatment	4.0 ± 2.3	3.2	4.1 ± 2.2	3.2	0.610^‡^
Change^*^	-60.7 ± 48.7	-60.7	-78.4 ± 51.8	-69.1	0.250^‡^
P values^†^	**<0.001^¶^**		**<0.001^¶^**		
NLR					
Pretreatment	3.43 ± 1.24	3.90	3.27 ± 1.05	3.30	0.649^§^
Posttreatment	1.95 ± 0.72	2.00	1.76 ± 0.64	1.50	0.380^§^
Change^*^	-1.48 ± 0.71	-1.41	-1.51 ± 0.89	-1.30	0.919^§^
P values^†^	**<0.001^#^**		**<0.001^#^**		
IG					
Pretreatment	0.04 ± 0.02	0.04	0.04 ± 0.02	0.03	0.989^‡^
Posttreatment	0.02 ± 0.01	0.01	0.02 ± 0.01	0.02	0.484^‡^
Change^*^	-0.02 ± 0.02	-0.02	-0.02 ± 0.02	-0.02	1.000^‡^
P values^†^	**0.002^¶^**		**<0.001^¶^**		

^*^Change from pretreatment to posttreatment values. ^†^Intragroup change. ^‡^Mann-Whitney U test; ^§^Independent samples t test; ^¶^Wilcoxon test. ^#^Paired samples t test. Abbreviations: CRP, C-reactive protein; ESR, erythrocyte sedimentation rate; fT3, free triiodothyronine; fT4, free thyroxine; IG, immature granulocytes; NLR, neutrophil-to-lymphocyte ratio; NSAIDs, nonsteroidal antiinflammatory drugs; TSH, thyroid stimulating hormone.

As shown in Table 4, no significant correlation was observed between pretreatment NLR and ESR values or between pretreatment IG and ESR values (p > 0.05 for both). In contrast, significant positive correlations were observed between pretreatment NLR and CRP values and between pretreatment IG and CRP values (p < 0.05 for both). No significant correlation was observed between posttreatment NLR and ESR values, posttreatment IG and ESR values, or posttreatment IG and CRP values (p > 0.05 for all). In contrast, a significant (p < 0.05) positive correlation was observed between posttreatment NLR and CRP values. Significant positive correlations were observed between pretreatment to posttreatment NLR change and ESR and CRP changes, as well as pretreatment to posttreatment IG change and CRP change (p < 0.05 for all). In contrast, no significant correlation was observed between pretreatment to posttreatment IG change and ESR change (Table 4).

**Table 4:** Correlation between erythrocyte sedimentation rate and C-reactive protein with neutrophil-to-lymphocyte ratio and immature granulocytes before and after treatment

		ESR	CRP
Pretreatment			
NLR	r	0.298	0.452
	p	0.058	**0.003**
IG	r	0.277	0.397
	p	0.080	**0.010**
Posttreatment			
NLR	r	0.254	0.428
	p	0.109	**0.005**
IG	r	-0.048	0.134
	p	0.763	0.405
Posttreatment change			
NLR	r	0.351	0.493
	p	**0.024**	**0.001**
IG	r	0.223	0.352
	p	0.161	**0.024**

Abbreviations: CRP, C-reactive protein; ESR, erythrocyte sedimentation rate; IG, immature granulocytes; NLR, neutrophil-to-lymphocyte ratio.

## DISCUSSION

Subacute thyroiditis is a painful inflammatory thyroid disease most commonly seen in patients aged 30-50 years (15). The mean age at diagnosis has been reported as 34 ± 17.8 years by Erdem and cols. (16), 35 ± 11.2 years by Alfadda and cols. (17), and 45.4 ± 9.7 years by Cappelli and cols. (18). In the present study, the mean age of the patients at diagnosis was 41.5 ± 8.6 years, which is consistent with literature reports.

Subacute thyroiditis is observed with a higher (3-5 times) frequency in women than men, with the women-to-men ratio ranging from 1.8-14.1:1 across studies (16,17). The ratio found in the present study (3.1:1), indicating a higher proportion of women to men, is consistent with literature findings.

Increased rates of subacute thyroiditis have been reported during or after COVID-19 (3,4). In the present study, 10 patients (24.4%) developed subacute thyroiditis after COVID-19 infection. Additionally, cases of subacute thyroiditis after COVID-19 mRNA vaccines have been reported in the literature (19). Two (4.9%) patients in the present study were diagnosed with subacute thyroiditis after receiving the BioNTech (mRNA) COVID-19 vaccine.

Both ESR and CRP are valuable tests in the diagnosis of subacute thyroiditis. These tests are not specific to a single disease, as they can be elevated in many conditions. Still, they are used for following up the clinical course of the disease in patients with subacute thyroiditis. In the later stages of the disease, ESR and CRP values decrease as inflammation reduces. In the present study, ESR and CRP values decreased in all patients.

The results of the present study indicate that IG and NLR values could be used as markers of treatment response in patients with subacute thyroiditis. Our study is the first to compare IG and NLR values in patients with this condition. Of note, several studies have examined NLR values in different thyroid diseases. Taiwanese researchers have shown that NLR is associated with the size of thyroid tumors and that a high preoperative NLR is associated with increased tumor size and increased risk of recurrence in patients with differentiated thyroid cancer (20).

The 5-year disease-free survival of stage 3-4 papillary thyroid cancer has been shown to be worse in patients with high compared with low NLR values (21). In a study by Aktas and cols., NLR values were higher in patients with Hashimoto’s thyroiditis than in healthy controls (22), which may be explained by lymphocytic infiltration of the thyroid gland.

Subacute thyroiditis is thought to result from a viral infection and a postviral inflammatory process. The present study found a significant decrease in posttreatment from pretreatment NLR values and high ESR and CRP values in the acute phase of the disease. Both ESR and CRP values are inflammatory markers of response to treatment. While CRP is one of the best-known inflammatory markers, NLR has been found to correlate with CRP levels (23,24).

The findings of a significant positive correlation between pretreatment and posttreatment NLR values and pretreatment and posttreatment CRP values in the present study are aligned with reports from the literature. Additionally, a significant positive correlation was observed between treatment and changes in NLR, ESR, and CRP values. In patients treated with NSAIDs and corticosteroids, posttreatment NLR values decreased significantly relative to pretreatment values. No difference in this regard was observed between treatment groups.

Increased differentiation of polymorphonuclear leukocytes in inflammatory and infectious conditions begins with the formation of IG. Thus, IG in peripheral blood indicates increased bone marrow activation. Technical advances in automated hematology analyzers now allow for easy determination of IG in routine CBC and the use of the results as an inflammatory biomarker. Compared with healthy individuals, those with sepsis or infection have significantly increased IG results (25-27). In the literature, IG values have been used as a marker of disease severity in patients with severe perforated acute appendicitis or necrotizing pancreatitis. It has also been shown that IG is a more effective and reliable early predictor of acute necrotizing pancreatitis compared with traditional inflammatory markers like leukocytes, NLR, and CRP. Furthermore, compared with other hematological parameters, IG is a more reliable marker in identifying acute appendicitis and determining its progression to complicated appendicitis in cases where the diagnosis of acute appendicitis is doubtful (28,29).

The present study investigated pretreatment to posttreatment changes in values of IG, a new inflammatory marker, in subacute thyroiditis. The results showed that IG decreased significantly after treatment relative to pretreatment values and indicated a significant positive correlation between IG and CRP changes. In both treatment groups (NSAID and corticosteroid), IG values decreased significantly after treatment relative to pretreatment values. Additionally, no difference in IG change was observed between treatment groups. When the groups treated with NSAID versus corticosteroids were compared, no significant difference was observed regarding pretreatment and posttreatment values of ESR, CRP, NLR, and IG. These findings indicate that IG and NLR may be used to assess treatment response in subacute thyroiditis and that the choice between treatment with NSAIDs or corticosteroids does not influence the inflammatory markers during follow-up.

In conclusion, early diagnosis and treatment of subacute thyroiditis are critical. Both IG and NLR – inflammatory markers easily detected in routine CBC tests – decreased significantly after treatment relative to pretreatment values, a finding that was observed in patients treated with NSAIDs or corticosteroids. Additionally, NLR changes correlated with ESR and CRP changes, while IG changes correlated only with CRP changes. These findings indicate that IG and NLR may be used as markers of treatment response in patients with subacute thyroiditis.
